# Genetic diversity of *Colletotrichum lupini* and its virulence on white and Andean lupin

**DOI:** 10.1038/s41598-021-92953-y

**Published:** 2021-06-29

**Authors:** J. A. Alkemade, M. M. Messmer, R. T. Voegele, M. R. Finckh, P. Hohmann

**Affiliations:** 1 Department of Crop Sciences, Research Institute of Organic Agriculture (FiBL), Frick, Switzerland; 2 Institute of Phytomedicine, University of Hohenheim, Stuttgart, Germany; 3 Department of Ecological Plant Protection, University of Kassel, Kassel, Germany

**Keywords:** Agricultural genetics, Genetic interaction, Genotype, Microbial genetics, Plant breeding, Sequencing, Fungi, Microbial genetics, Pathogens, Plant breeding

## Abstract

Lupin cultivation worldwide is threatened by anthracnose, a destructive disease caused by the seed- and air-borne fungal pathogen *Colletotrichum lupini*. In this study we explored the intraspecific diversity of 39 *C. lupini* isolates collected from different lupin cultivating regions around the world, and representative isolates were screened for their pathogenicity and virulence on white and Andean lupin. Multi-locus phylogeny and morphological characterizations showed intraspecific diversity to be greater than previously shown, distinguishing a total of six genetic groups and ten distinct morphotypes. Highest diversity was found across South America, indicating it as the center of origin of *C. lupini*. The isolates that correspond to the current pandemic belong to a genetic and morphological uniform group, were globally widespread, and showed high virulence on tested white and Andean lupin accessions. Isolates belonging to the other five genetic groups were mostly found locally and showed distinct virulence patterns. Two highly virulent strains were shown to overcome resistance of advanced white lupin breeding material. This stresses the need to be careful with international seed transports in order to prevent spread of currently confined but potentially highly virulent strains. This study improves our understanding of the diversity, phylogeography and pathogenicity of a member of one of the world’s top 10 plant pathogen genera, providing valuable information for breeding programs and future disease management.

## Introduction

The fungal genus *Colletotrichum* contains many important plant pathogenic species that cause anthracnose and other pre- and post-harvest diseases in a wide variety of hosts^1–4^. Among potential hosts are important fruit, cereal and legume crops such as strawberry^5^, maize^6^ and soybean^7,8^. Besides being of economic importance, *Colletotrichum* spp. have been widely used as model species to study plant-fungus interactions because of the diversity of lifestyles within this genus^9–12^. *Colletotrichum* is listed in the top 10 of most important fungal plant pathogens worldwide^13^. Within the genus, members of the *Colletotrichum acutatum* species complex are notorious and cause disease in many important crops^14,15^. The most important morphological characteristic for members of this species complex are the acute ends of its conidia^14^. Discrimination of *Colletotrichum* species solely based on morphological traits, however, is deemed unreliable due to the few and highly variable characteristics, the strong influence of environmental conditions and the high overlap between species^16^. Therefore, a polyphasic approach, combining morphological and genetic data is recommended^17,18^. Multi-locus phylogeny revealed a high diversity within the *C. acutatum* species complex, showing at least 32 different species divided among five clades^14^. Although many species within the *C. acutatum* species complex have a broad host range, *Colletotrichum lupini*, belonging to clade 1, appears to be highly host specific on lupins (*Lupinus*)^19,20^.

**Figure 1:**
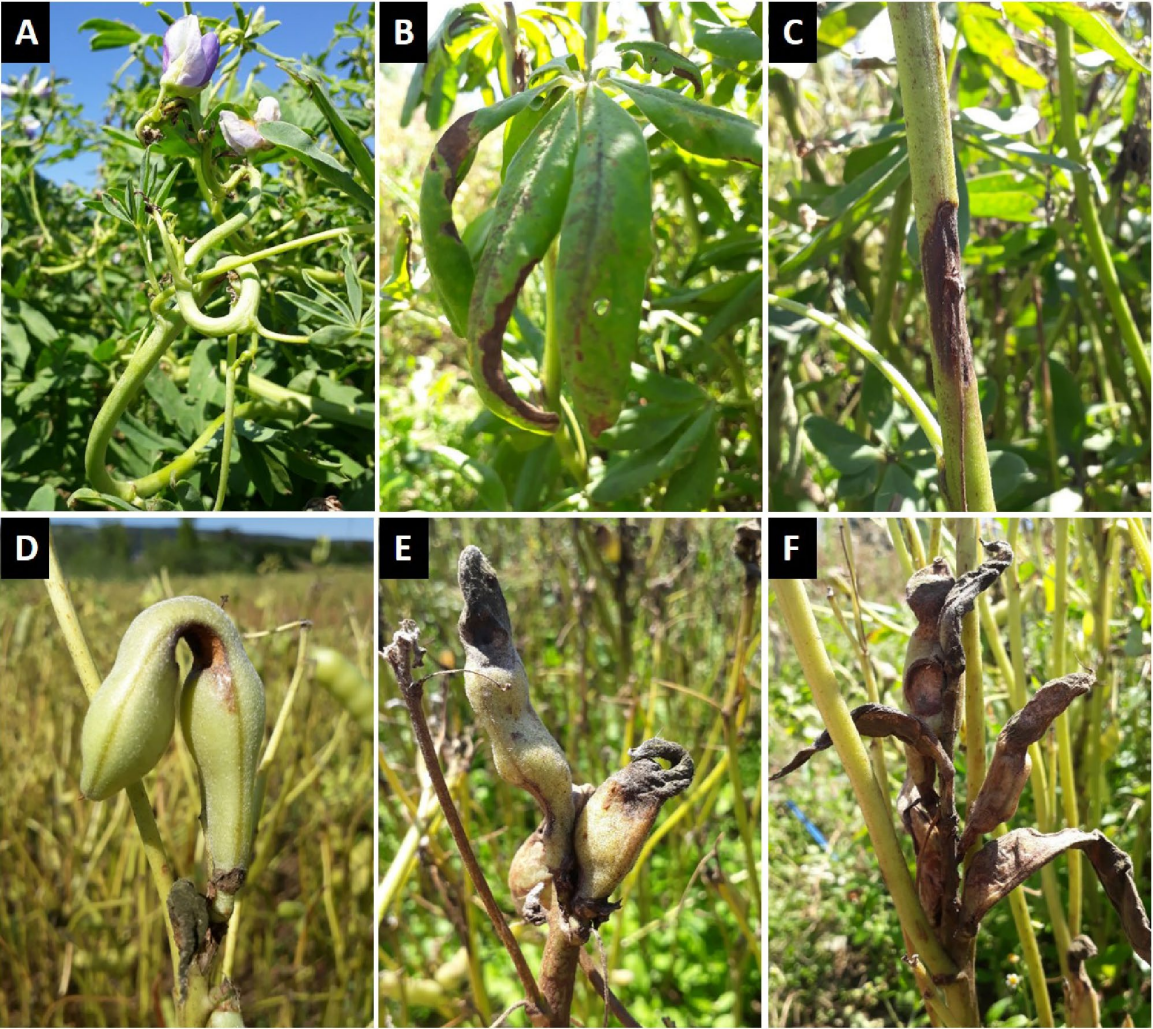
Symptoms on lupin tissue associated with *Collletotrichum lupini*. (**A**) typical stem twisting (*Lupinus mutabilis*); (**B**) on the leaves (*L. albus*); (**C**) on the main stem (*L. albus*); (**D**–**F**) on the pods (*L. albus*). Photos by Alkemade JA.

Lupin anthracnose caused by *C. lupini* is the most important disease in lupin cultivation worldwide, affecting all economically important lupin species such as blue (*Lupinus angustifolius* L.), white (*L. albus* L.), Andean (*L. mutabilis* Sweet.), yellow (*L. luteus* L.) and ornamental lupin (*L. polyphyllus* Lindl.)^20^. The disease was first reported in 1912 in Brazil^21^, but the fungal pathogen was identified much later^22^. A first outbreak was reported in the 1940–1950s in North America and was followed by a more severe and globally widespread outbreak around the 1980s which is still persisting until this day^20^. The disease is mainly dispersed via seeds, facilitating rapid spread through international seed transports, and within the crop by rain splash during the growing season^23^. Even low amounts of initial inoculum can cause total yield losses making this disease highly destructive^24,25^. Typical symptoms are stem twisting and necrotic lesions on stems and pods (Fig. 1)^26^. Current disease management is focused on planting certified disease-free seed and chemical protection^23,27^. However, crop resistance could offer a more sustainable alternative. In blue lupin, anthracnose resistance is controlled by single resistance genes^28–30^, whereas in white, Andean and yellow lupin no such single gene resistance is known and the observed quantitative resistance is considered to be polygenic^31–33^. The increasing demand for plant-based protein is renewing the interest for lupins as a high quality protein crop^34–36^, the current anthracnose pandemic, however, severely hampers cultivation.

The pathogen was first described as *Gloesporium lupini*, followed by *C. gloeosporioides* and *C. acutatum* until it was fully described as *C. lupini*^14,37,38^. Currently two genetic groups (I and II) are distinguished within *C. lupini* based on vegetative compatibility groups (VCG)^38^, the ITS (internal transcribed spacer) region^37^ and multi-locus phylogeny of the ITS, GAPDH (glyceraldehyde-3-phosphate dehydrogenase), CHS-1 (chitin synthase), HIS3 (histone), ACT (actin), TUB2 (β-tubulin 2), HMG (HMG box region) and APN/MAT1 (Apn2-Mat1-2-1 intergenic) loci^39^. The TUB2 and GAPDH loci were shown to be the most informative within the *C. acutatum* species complex and APN/MAT1 the most informative within *C. lupini*, whereas classification based on the ITS region can be problematic due to low resolution within the complex^14,39^. Although only two groups within *C. lupini* have been distinguished, with most of the reported strains belonging to group II^39^, intraspecific diversity is thought to be greater as a high diversity was found in a Chilean *C. lupini* collection using random amplified polymorphic DNA (RAPD) markers^40^ and a distinct lupin infecting *C. acutatum* group was identified in Ecuador based on the ITS region^41^. This suggests that highest intraspecific diversity is found in South America, which is believed to be the center of origin of members belonging to clade 1 of the *C. acutatum* species complex^10,15^.

The overall aim of this study was to assess a worldwide collection of lupin-infecting *Colletotrichum* isolates through (i) multi-locus phylogeny, (ii) morphology and (iii) virulence on white and Andean lupin. Insights into *C. lupini* diversity, phylogeography and plant-*C. lupini* interactions will improve our understanding of the current lupin anthracnose pandemic and support future disease management strategies and lupin breeding programs.

## Results

### *Colletotrichum lupini* comprises of six genetic groups supported by morphology

**Figure 2:**
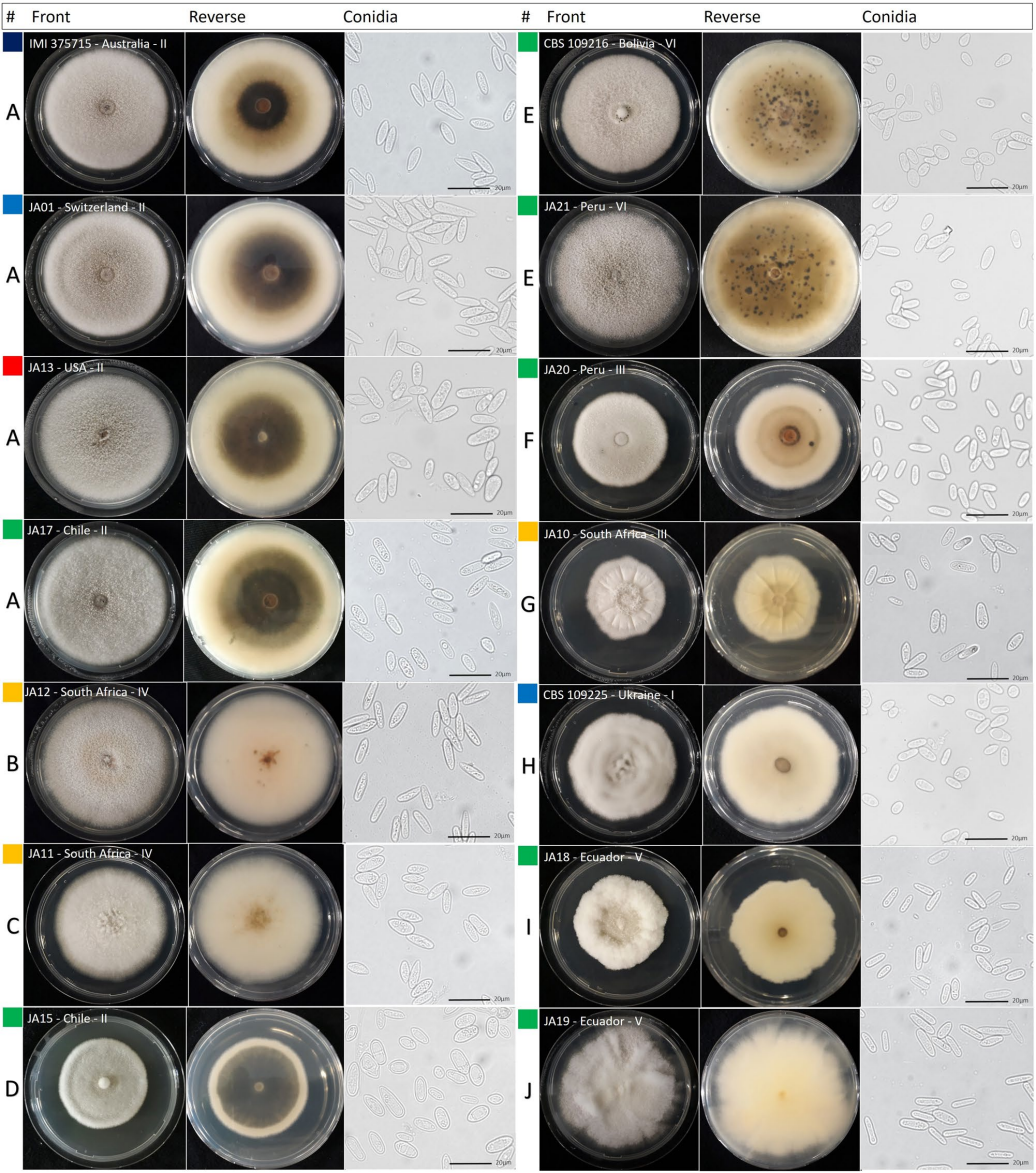
*Colletotrichum lupini* morphology. Capital letters (A–J) indicate the different morphology types based on conidia shape and size and colony growth rate and morphology (see Table 2). Strain codes are followed by country of origin and roman numbers (I–VI) indicate genetic groups. Plates show the front and reverse of 14 day old colonies on PDA. Scale bars indicate 20 µm. Colors indicate strain origin: blue = Europe, green = South America, red = North America, orange = Southern Africa, dark blue = Australia.

From the 50 sequenced isolates, 39 belonged to *C. lupini* (Table 1). A globally representative subset of 28 *C. lupini* isolates was characterized based colony morphology (form, aerial mycelium, margin type and color of the reverse side) and 18 of those were further characterized for growth rate and conidial shape and size, revealing ten distinct morphotypes (A–J; Fig. 2, Table 2, Supplementary Figs. S1, S2). Despite certain variability, all observed conidia shared features typical for *C. lupini* (hyaline, smooth-walled, aseptate, straight and with one acute end) as described by Damm et al*.*^14^. Morphotype A was the most common and was observed for isolates from across the world (Europe, Australia, North- and South America), all belonging to genetic group II. Morphotypes B, C and G were observed for isolates from South Africa and morphotypes D, E, G, I and J were observed for isolates from South America.

**Table 1:** Isolation details and GenBank accessions of *Colletotrichum* strains used in this study.

Strain^a^	Alternative code(s)	Species	Host	Origin	Year	GenBank no.^b^			
						ITS	GAPDH	TUB2	APN/MAT1
**JA01**		*Colletotrichum lupini*	*Lupinus albus*	Switzerland, Melikon	2018	MT741840	**MW342515**	**MW342537**	**MW342559**
**JA02**		*C. lupini*	*L. albus*	Switzerland, Feldbach	2019	**MW342494**	**MW342516**	**MW342538**	**MW342560**
**JA03**		*C. lupini*	*L. albus*	Germany, Hattenhofen	2019	**MW342495**	**MW342517**	**MW342539**	**MW342561**
**JA04**		*C. lupini*	*L. albus*	Germany, Witzenhausen	2018	**MW342496**	**MW342518**	**MW342540**	**MW342562**
**JA05**		*C. lupini*	*L. albus*	Germany, Westerau	2018	**MW342497**	**MW342519**	**MW342541**	**MW342563**
**JA06**		*C. lupini*	*L. albus*	Russia, Saint Petersburg	2018	**MW342498**	**MW342520**	**MW342542**	**MW342564**
**JA07**	BRIP 63850, WAC 12994	*C. lupini*	*L. angustifolius*	Australia, WA, Dongara	2004	**MW342499**	**MW342521**	**MW342543**	**MW342565**
**JA08**	BRIP 63851, WAC 12995	*C. lupini*	*L. luteus*	Australia, WA, Mingenew	2004	**MW342500**	**MW342522**	**MW342544**	**MW342566**
**JA09**	BRIP 63857, WAC 13001	*C. lupini*	*L. albus*	Australia, WA, Yandanooka	2004	**MW342501**	**MW342523**	**MW342545**	**MW342567**
**JA10**	CMW 9930, SHK 788	*C. lupini*	*L. albus*	South Africa, Bethlehem	1994	**MW342502**	**MW342524**	**MW342546**	**MW342568**
**JA11**	CMW 9931, SHK 1033	*C. lupini*	*L. albus*	South Africa, Stellenbosch	1995	**MW342503**	**MW342525**	**MW342547**	**MW342569**
**JA12**	CMW 9933, SHK 2148	*C. lupini*	*L. albus*	South Africa, Malmesbury	1999	**MW342504**	**MW342526**	**MW342548**	**MW342570**
**JA13**		*C. lupini*	*L. mutabilis*	USA, Florida, Martin County	2013	**MW342505**	**MW342527**	**MW342549**	**MW342571**
**JA14**		*C. lupini*	*L. hartwegii*	USA, Florida, Martin County	2013	**MW342506**	**MW342528**	**MW342550**	**MW342572**
**JA15**	A-02	*C. lupini*	*L. albus*	Chile, Cajón	2009	**MW342507**	**MW342529**	**MW342551**	**MW342573**
**JA16**	A-10	*C. lupini*	*L. angustifolius*	Chile, Cajón	2009	**MW342508**	**MW342530**	**MW342552**	**MW342574**
**JA17**	A-24	*C. lupini*	*L. albus*	Chile, Temuco	2015	**MW342509**	**MW342531**	**MW342553**	**MW342575**
**JA18**	Lup1	*C. lupini*	*L. mutabilis*	Ecuador, Juan Montalvo	2007	**MW342510**	**MW342532**	**MW342554**	**MW342576**
**JA19**	Lup18	*C. lupini*	*L. mutabilis*	Ecuador, Pujili	2007	**MW342511**	**MW342533**	**MW342555**	**MW342577**
**JA20**		*C. lupini*	*L. mutabilis*	Peru, Carhuaz	2019	**MW342512**	**MW342534**	**MW342556**	**MW342578**
**JA21**		*C. lupini*	*L. mutabilis*	Peru, Carhuaz	2019	**MW342513**	**MW342535**	**MW342557**	**MW342579**
**JA22**		*C. lupini*	*L. mutabilis*	Peru, Carhuaz	2019	**MW342514**	**MW342536**	**MW342558**	**MW342580**
**CBS 109216**	BBA 63879	*C. lupini*	*L. mutabilis*	Bolivia		JQ948156	JQ948486	JQ949807	**MW342581**
**CBS 109221**	BBA 70352, RB172	*C. lupini*	*L. albus*	Germany		JQ948169	JQ948499	JQ949820	MK478328
**CBS 109225**	BBA 70884	*C. Lupini*	*L. albus*	Ukraine		JQ948155	JQ948485	JQ949806	MK478329
CBS 109226	RB121, IMI 504884, HY09, BBA 71249	*C. lupini*	*L. albus*	Canada, Nova Scotia		JQ948158	JQ948488	MK478189	MK478316
CBS 509.97	RB235, LARS 178	*C. lupini*	*L. albus*	France		JQ948159	JQ948489	JQ949810	MK478355
**IMI 375715**	96A4	*C. lupini*	*L. albus*	Australia, WA, Perth	1997	JQ948161	JQ948491	JQ949812	MK478341
RB020	PT30	*C. lupini*	*L. albus*	Portugal, Azores	1999	MK463722	KM252117	MK478186	MK478308
RB042	CBS 129944, CMG12	*C. lupini*	*Cinnamonium zeylanicum*	Portugal, Lisbon	1996	MH865693	JQ948508	JQ949829	MK478310
RB116	CSL 1294	*C. lupini*	*L. polyphyllus*	UK, York		MK463723	KM252194	KM251944	MK478313
RB122	BBA 71310, C3	*C. lupini*	*L. luteus*	Poland		MK463726	MK463750	MK478190	MK478317
RB123	IMI 504885, SHK788	*C. lupini*	*L. albus*	South Africa, Bethlehem	1994	MK463727	MK463751	MK478191	MK478318
RB124	BBA 70555	*C. lupini*	*L. albus*	Chile		MK463728	MK463752	MK478192	MK478319
RB125	CBS 109224, BBA 70399	*C. lupini*	*L. albus*	Austria		JQ948172	JQ948502	JQ949823	MK478320
RB127	PT702	*C. lupini*	*Olea europaea*	Spain		MK463729	MK463753	MK478193	MK478321
RB147	IMI 350308	*C. lupini*	*Lupinus* sp.	UK, Kent	1991	MK463730	KM252203	KM251951	MK478322
**RB221**	IMI 504893	*C. lupini*	*Lupinus* sp.	France, Brittany	2016	MK463733	MK463756	MK478196	MK478345
**RB226**		*C. lupini*	*Lupinus* sp.	France, Brittany	2016	MK463738	MK463761	MK478201	MK478350
CBS 129814	T.A6	*C. tamarilloi*	*Solanum betaceum*	Colombia, Gundinamarca	2012	JQ948184	JQ948514	JQ949835	**MW342584**
CBS 129955	RB018, Tom-12	*C. tamarilloi*	*Solanum betaceum*	Colombia, Antioquia, Santa Rosa	1998	JQ948189	JQ948519	JQ949840	MK478307
CBS 211.78	IMI 309622, RB184	*C. costaricensis*	*Coffea* sp.	Costa Rica, Turrialba		JQ948181	JQ948511	JQ949832	MK478333
CBS 134730	RB237	*C. melonis*	*Malus domestica*	Brazil, Rio Grande do Brazil		KC204997	KC205031	KC205065	MK478357
IMI 304,802	RB216	*C. cuscutae*	*Cuscuta* sp.	Dominica		JQ948195	JQ948525	JQ949846	MK478340
IMI 384185	CPC 18937, RB218	*C. paranaense*	*Caryocar brasiliense*	Brazil		JQ948191	JQ948521	JQ949842	MK478342
CBS 130239	Frag NL-1	*C. nymphaeae*	*Fragaria x ananassa*	The Netherlands	2011	JQ948250	JQ948580	JQ949901	**MW342583**
IMI 360928	CPC 18926, RB163	*C. nymphaeae*	*Fragaria x ananassa*	Switzerland, Zürich	1993	JQ948243	JQ948573	JQ949894	MK478326
CBS 122122	BRIP28519, RB179	*C. simmondsii*	Carica papaya	Australia	1987	JQ948276	JQ948606	JQ949927	MK478332
CBS 369.73	NRCC 10081	*C. acutatum*	*L. angustifolius*	New Zealand, Kumeu	1968	JQ948350	JQ948681	JQ950001	**MW342582**
CBS 370.73	NRCC 10088, RB187	*C. acutatum*	*Pinus Aridata*	New Zealand, Tokoroa	1965	JQ948351	JQ948682	JQ950002	MK478335

*JA* strains from the FiBL culture collection characterized in this study, *RB* personal collection of Riccardo Baroncelli described in Dubrulle et al*.*^39^, *CBS* collection of the Westerdijk Fungal Biodiversity Institute, Utrecht, The Netherlands, *IMI* Culture collection of CABI Europe UK Centre, Egham, UK, *ITS* internal transcribed spacers 1 and 2 together with 5.8S nrDNA, *GAPDH* glyceraldehyde-3-phosphate dehydrogenase, *TUB2* β-tubulin 2, *APN/MAT1* Apn2-Mat1-2-1 intergenic.

Codes in bold were used for morphology analysis in this study.

Accession numbers in bold are newly determined in this study.

**Table 2:** Growth rate, conidial size and shape, and colony morphology for the different morphotypes observed within *Colletotrichum lupini*.

Strain	Morphotype	Genetic group	Growth rate (mm/day)^a^	Conidia L × W (µm)^a^	Conidia shape^bc^	Colony morphology^c^
IMI 375715, JA01, -06, -07, -13, -16, -17	A	II	6.2 ± 0.1	12 ± 2.1 × 4 ± 0.7	Cylindrical to elliptical, occasionally clavate	Flat, circular, with entire margins, white-greyish cottony aerial mycelium, pale to orange on reverse, dark in center
JA12	B	IV	5.6 ± 0.1	13.3 ± 1.4 × 3.4 ± 0.5	Cylindrical to elliptical, occasionally clavate	Flat, circular, with entire margins, white-brownish cottony aerial mycelium, pale on reverse
JA11	C	IV	5.5 ± 0.1	12 ± 1.7 × 4.5 ± 0.7	Cylindrical to elliptical, occasionally clavate	Flat, circular, slightly filiform margins, white-greyish cottony aerial mycelium, pale on reverse, orange in center
JA15	D	II	5 ± 0	9.7 ± 2.4 × 4.2 ± 1.0	Cylindrical, occasionally roundish	Flat, circular, with entire margins, white-greyish cottony aerial mycelium, dark on reverse, pale at margins
CBS 109216, JA21, -22	E	VI	5.4 ± 0.3	8.5 ± 2.1 × 3.5 ± 0.7	Cylindrical to clavate	Flat, circular, with entire margins, white-greyish cottony aerial mycelium, pale orange on reverse with black dots
JA20	F	III	4.2 ± 0.3	8.7 ± 1.1 × 3.2 ± 0.6	Cylindrical, occasionally clavate	Flat, circular, with entire margins , sparse white-greyish aerial mycelium, pale on reverse
JA10	G	III	4.9 ± 0.2	9.2 ± 1.7 × 3.6 ± 0.7	Cylindrical to elliptical, occasionally clavate	Irregular and radially sulcate with aerial mycelia growth in the center, pale on reverse
CBS 109225	H	I	5.2 ± 0.1	8.5 ± 1.7 × 3.8 ± 0.8	Cylindrical to clavate	Slightly irregular and thickly covered with wooly white-greyish aerial mycelia, pale on reverse
JA18	I	V	4.1 ± 0	10 ± 1.8 × 2.9 ± 0.7	Cylindrical	Irregular, wooly white areal mycelia on the margins, pale on reverse
JA19	J	V	6 ± 0.2	12.1 ± 1.8 × 2.4 ± 0.7	Cylindrical	Irregular, white-greyish wooly aerial mycelium, pale on reverse with occasional black/orange dots

*L* length, *W* width.

^a^Mean ± SD, see also Supplementary Fig. S2.

^b^Observed conidia were rather variable in shape and size, but all conidia were hyaline, smooth-walled, aseptate, straight, with one end round and one end acute as described for *Colletotrichum lupini* in Damm et al*.*^14^.

^c^See also Fig. 2.

**Figure 3:**
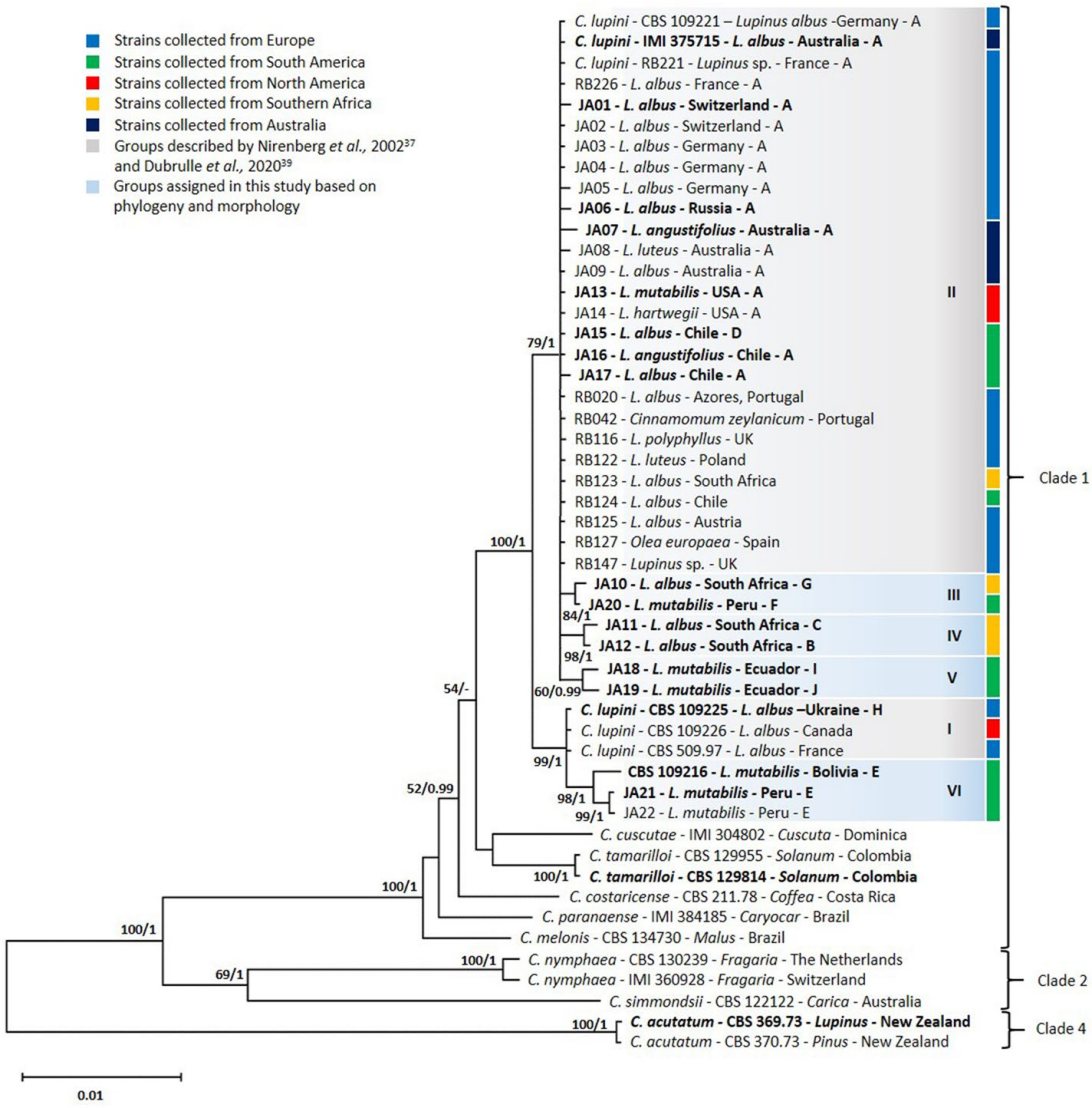
Multi-locus phylogeny of *Colletotrichum lupini*. Bayesian analysis tree inferred from the combined ITS, TUB2, GAPDH and APN/MAT1 sequence datasets of 50 *Colletotrichum* strains used in this study. Bootstrap support values (> 50) and Bayesian posterior probabilities (> 0.95) are given at each node. The tree is rooted to *C. acutatum* (CBS 369.73 and CBS 370.73). Strain codes are followed by host, country of origin and morphology (A–J). Grouping (I–VI) is based on phylogeny and morphology. Strains used for virulence assays are highlighted in bold. Clades indicate the different clades within the *C. acutatum* species complex.

Multi-locus phylogenetic analyses of 50 *Colletotrichum* isolates identified six distinct genetic groups within *C. lupini* (I–VI; Fig. 3, Supplementary Fig. S3). The combined sequence dataset contained 2251 characters (ITS: 1–496, GAPDH: 497–745, TUB2: 746–1200, APN/MAT1: 1201–2251) including alignment gaps. The APN/MAT1 locus showed the highest variability across the nucleotide data set, with 75.8% conserved sites for the whole data set (including out-groups) and 97.4% within *C. lupini* (Supplementary Table S1). The TUB2 and GAPDH loci showed 89.9% and 81.1% identical sites for the entire dataset and 97.8% and 98.4% identity within *C. lupini*, respectively. The ITS region showed the lowest variability with 97% identical sites across the whole dataset and 99.2% within *C. lupini.* As shown in Fig. 3, most *C. lupini* strains clustered with a high bootstrap support (BS) value of 79 and posterior probability (PP) of 1 with reference strains representing genetic group II (CBS 109221, IMI 375715 and RB221). Strains within group II showed a high identity among each other (> 99.9%) and showed morphotype A, except for Chilean strain JA15 showing morphotype D (Fig. 2). South African strain JA10 and Peruvian strain JA20, with morphotypes G and F, respectively, clustered together with a BS of 84 and PP of 1, forming a highly supported group (III). South African strains JA11 and JA12, with morphotypes C and B, respectively, clustered together with a BS of 98 and PP of 1, forming a highly supported group (IV). Ecuadorian strains JA18 and JA19 with distinct morphotypes I and J, respectively, showed 99.7% identity with reference strains of group II and clustered together with a BS of 60 (Fig. 3, Supplementary Fig. S3) and a PP of 1 in (Fig. 3), forming a distinct group (V). The reference strains for group I (CBS 109225 with morphotype H, CBS 109226 and CBS 509.97) are clustered together with a BS of 99 and PP of 1 and show 100% identity with each other and 99.6% identity with reference strains of group II. South American strains JA21, JA22 and CBS 109216, with morphotype E, cluster together with a BS of 98 and PP of 1 (Fig. 3) and a BS of 54 (Supplementary Fig. S3) forming a highly supported group (VI). JA21 and JA22 showed 99.8% and CBS 109216 showed 99.7% identity with reference strains of group I and 99.4% and 99.2% identity with references strains of group II, respectively.

### Distinct virulence patterns on white and Andean lupin

**Figure 4:**
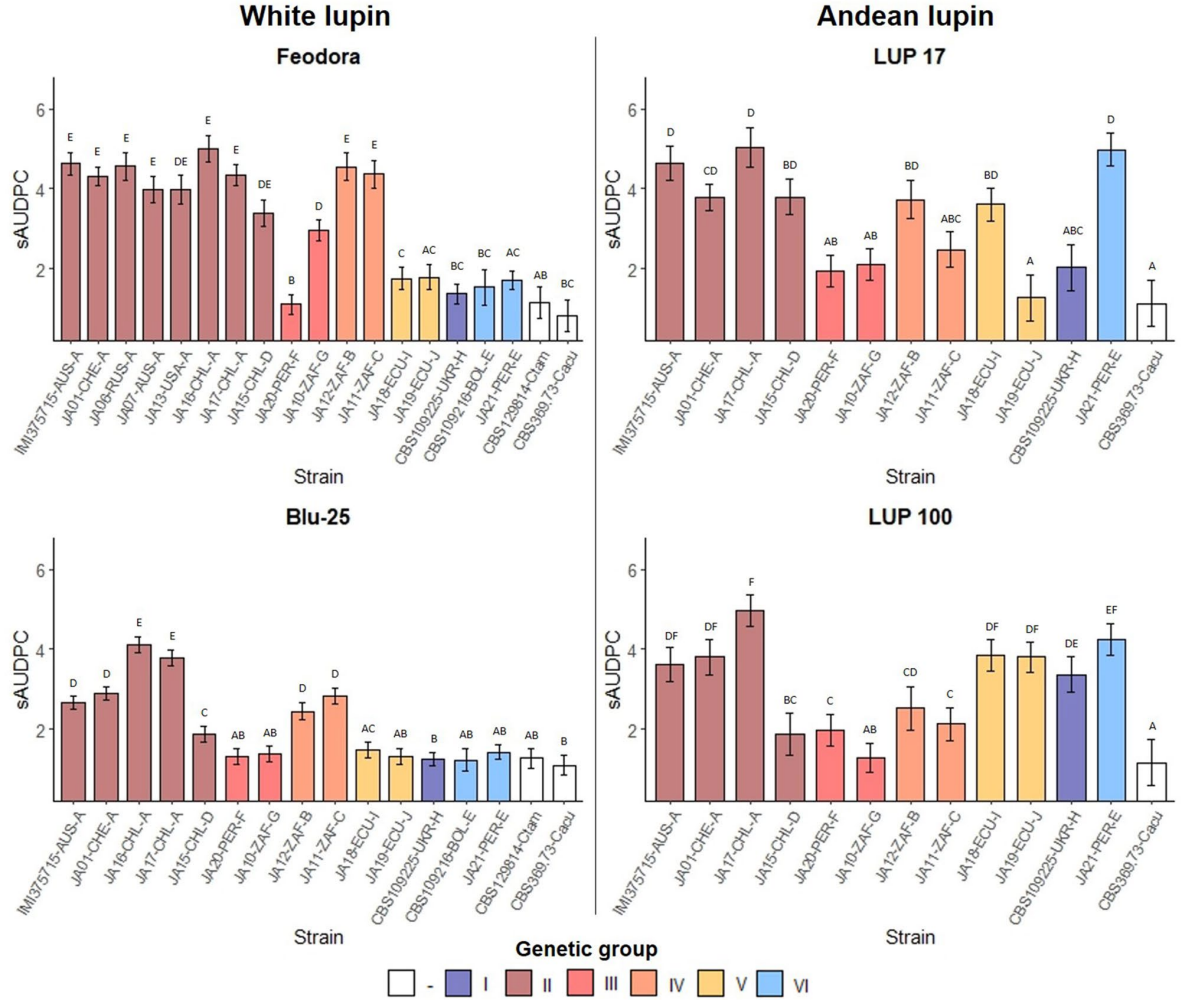
Virulence of *Colletotrichum lupini* strains on white (*Lupinus albus)* and Andean lupin (*L. mutabilis*). Anthracnose severity is expressed in standardized area under the disease progress curve (sAUDPC) and estimated means are shown. Strain codes are followed by abbreviated country of origin and morphotype (A–J). Different capital letters above bars indicate significant differences between strains (Tuckey-HSD, p < 0.05). Error bars indicate the standard error of the estimated mean.

Virulence assays performed on two white lupin (*L. albus* L.) accessions (Feodora and Blu-25) and two Andean lupin (*L. mutabilis* Sweet.) accessions (LUP 17 and LUP 100) with strains representing the different morphotypes and genetic groups indicated in Fig. 3, revealed strong strain (p < 0.0001), lupin species (p < 0.0001) and strain × lupin species interaction effects (p < 0.0001). A strong accession effect was found within white lupin (p < 0.0001), whereas for Andean lupin there was no significant accession effect (p = 0.43). Strain (p < 0.0001) and strain × accession (p < 0.0001) interaction effects were found for both species. Strains belonging to genetic group II with morphotype A, caused severe disease on white lupin accession Feodora and both Andean lupin accessions (Supplementary Fig. S4), showing standardized area under the disease progress curve (sAUDPC) means ranging from 3.95 to 5 (Fig. 4). On the more tolerant white lupin accession Blu-25, sAUDPC means for strains of group II with morphology A were more variable, with JA01 and IMI 375715 showing moderate (2.7–2.9) and Chilean strains JA16 and 17 showing high (3.8–4.1) virulence. Chilean strain JA15, also belonging to genetic group II but with a different morphology (D), caused low disease on LUP 100 and Blu-25 (1.9), showing a different virulence spectrum compared to the other tested strains of genetic group II. South African strains JA11 and JA12, belonging to genetic group IV with morphotypes C and B, respectively, showed a similar virulence spectrum on white lupin as strains of group II. JA10 and JA20, representing group III and morphotype G and F, respectively, were overall avirulent (< 2), with the exception of JA10 on Feodora, showing moderate virulence (2.95). Peruvian strain JA21, representing genetic group VI and morphotype E, caused low disease on white lupin (1.4–1.8), but severe disease on Andean lupin (4.25–5). A similar observation was found for the two Ecuadorian strains JA18 and JA19 of genetic group V and morphotypes I and J, respectively. These two strains caused low disease on white lupin and high disease on Andean lupin LUP 100. On Andean lupin LUP 17, however, a severe disease phenotype was only found for JA18 (3.6), whereas JA19 barely caused any disease symptoms (1.25). Similar to the observations for JA19, the Ukrainian strain CBS 109225 (genetic group I, morphotype H) caused severe disease on Andean lupin LUP 100 (3.36) and low disease on Andean lupin LUP 17 and white lupin (1.2–2). The *C. tamarilloi* and *C. acutatum* strains were avirulent across the lupin accessions (< 1.26).

## Discussion

This study compared 39 *C. lupini* and 11 *Colletotrichum* spp. isolates collected from across the world to explore intraspecific diversity of *C. lupini* and to better understand the dynamics of the current lupin anthracnose pandemic and potential implications of further migrations of distinct pathogenic strains. Based on multi-locus phylogeny supported by isolate morphology, we identified four distinct genetic groups additional to previously described genetic groups I and II. Highest intraspecific diversity was identified among *C. lupini* isolates collected from across the South American Andes region. This is in line with reports of Falconí et al*.*^41^ and Riegel et al*.*^40^ showing high diversity in Ecuador and Chile, respectively. In those regions, Andean lupin has been cultivated for more than 2000 years^42^ growing alongside numerous wild lupin species^43^. Isolates collected in South Africa showed a distinct morphology and virulence spectrum, indicating higher diversity than previously shown^44^. Although lupins form a significant part of the local agriculture and have been researched there since at least 1897^45^, they are not native to South Africa and lupin anthracnose was not reported in South Africa until 1993^46^. Taking into account the relatively recent reports of anthracnose in South Africa, the low diversity in Europe and Australia and the center of origin for species within clade 1 of the *C. acutatum* species complex being in South America^10,15^, we consider the South American Andes to be the center of origin of *C. lupini*.

The majority of the *C. lupini* isolates (26 out of 39) belong to the highly virulent genetic group II, showing morphotype A, and were collected in Europe, Australia, South Africa, the USA and Chile. This result confirms previous reports classifying most *C. lupini* strains from across the world in the same genetic group^14,39,47–49^. The low genetic diversity among strains of group II, the uniform morphology and non-observed sexual morph^14^ indicates clonality as suggested by Talhinhas et al*.*^20^. Pathogenicity of group II strains has also been shown on blue^28^, yellow^32^ and various other lupin species across the world^20^, indicating a broad host range within the genus *Lupinus*. Reports from South Korea and China indicate that group II strains also cause disease in those regions^50,51^, highlighting that these strains are globally widespread and are the cause of the current anthracnose pandemic in lupin. The group II strain RB221 can be used as reference, as it is now fully sequenced^52^ and tested on both Andean and white lupin^53^.

The stem-wound inoculation assay used in this study was previously described to be highly reproducible and strongly correlated to field performance under natural infection pressure^26^. In the present study, virulence assays based on stem-wounding showed strong strain x accession interaction effects for white and Andean lupin, suggesting a strain-dependent host spectrum and the existence of different physiological races within *C. lupini*. Similar observations were described by Falconí et al*.*^41^, showing a *C. lupini* strain x Andean lupin accession interaction effect. The existence of physiological races has been observed for various *Colletotrichum* species, such as for *C. lindemuthianum* on common bean^54^, *C. sublineola* on sorghum^55^ and *C. truncatum* on lentil^56^, but, in general, this is not common within the genus *Colletotrichum*. The similar virulence levels of isolates belonging to group II observed on Andean and white lupin accessions are in line with Alkemade et al*.*^26^, in which equal virulence was observed for IMI 375715 (Australia) and JA01 (Switzerland) when inoculated on six different white lupin accessions. However, an exception within group II is Chilean strain JA15, which, besides having a distinct morphology, was less virulent on Andean lupin LUP 100 and white lupin Blu-25. Further, Chilean strains JA16 and JA17 (also group II) overcame resistance of the resistant advanced breeding line Blu-25, which has been specifically bred for anthracnose resistance in Chile and was shown resistant under Swiss field conditions^26^. These results indicate that new introductions of highly virulent foreign strains can have severe consequences as seen for many other crops^57–59^ and it should be investigated if this high virulence is also affecting other resistant (white) lupin material^26,31,60^. Although disease development after stem-wounding of seedlings correlated strongly to field disease scores of mature plants^26^, we cannot exclude the possibility that conclusions drawn on virulence level might differ for secondary infection processes (e.g. via rain splash).

This study provides first solid evidence that, based on multi-locus phylogeny and morphology, genetic diversity within *C. lupini* is higher than previously shown. High-resolution genome-wide sequencing and an increased sampling density from especially the South American Andes region are now necessary to increase genetic resolution and to better understand *C. lupini* phylogeny and phylogeography. This could provide the basis for in-depth comparative genomic studies to identify effector gene clusters within the *C. lupini* genome. This study confirms that the current lupin anthracnose pandemic is caused by a genetically uniform group of highly virulent strains. The identification of strains with an increased virulence on tolerant white lupin breeding material and the observation of strain-specific virulence patterns should be taken into account in lupin resistance breeding programs. Due to its seed-borne nature, caution should be taken when importing seeds, especially from South America, to prevent further introductions of potentially virulent strains across the world.

## Methods

### Fungal and plant material

A diverse collection of 39 *Colletotrichum lupini* and 11 closely related *Colletotrichum* spp. isolates, originating from Europe, Australia, Southern Africa and South and North America, was analyzed (Table 1). Nine isolates were collected from symptomatic lupin plants in this study, whereas the rest of the isolates was already identified as *C. lupini* or as other members of the *C. acutatum* species complex representing clades 1, 2 and 4. The *C. lupini* strains CBS 109225 (Ukraine), CBS 509.97 (France) and CBS 109226 (Canada) were chosen as reference for genetic group I, strains CBS 109221 (Germany), IMI 375715 (Australia) and RB221 (France) served as reference for genetic group II and the *C. acutatum* strains CBS 369.73 and CBS 370.73 were used as outgroup in the phylogenetic analysis. Inoculations were performed on two white lupin (*Lupinus albus* L.) accessions: Feodora (susceptible; breeder: Jouffrai Drillaud, France) and Blu-25 (tolerant; breeder: Semillas Baer, Chile), and two Andean lupin (*L. mutabilis*) accessions: LUP 17 and LUP 100 (genebank: IPK, Germany). Plant material can be requested at mentioned breeders and genebanks, who performed formal identification and gave permission to use the material for research purposes. The experimental research of the plant material used in this study complies with relevant institutional, national, and international guidelines and legislation.

### Fungal isolation and culture conditions

Symptomatic (dried) lupin stem or pod tissue (Fig. 1) of 1–3 cm was surface sterilized (after rehydration in sterile ddH_2_O for dried samples) for 5 s with 0.25% sodium hypochlorite solution and rinsed thrice for 5 s in sterile ddH_2_O. Thin slices of 1 mm were cut and placed on PDA (potato dextrose agar, Carl Roth, Karlsruhe, Germany) amended with Tetracycline (0.02 g/l, Carl Roth) for 3 to 4 days at 22 °C in the dark. Single cultures were selected and grown on fresh PDA plates amended with Tetracycline for 4 to 6 days at 22 °C in the dark and suspected *Colletotrichum* species were sub-cultured. Single spore cultures were obtained and transferred to PDA and maintained at 22 °C in the dark as working cultures and stored at − 80 °C in 25% glycerol for long-term storage.

### Morphology

A globally representative subset of 28 *C. lupini* isolates was characterized based on colony morphology (form, aerial mycelium, margin type and color of the reverse side). From those, a subset of 18 isolates was further characterized for growth rate (mm/day), and conidial shape and size^19^. Isolates were subcultured by placing a droplet of 5 μl spore suspension in the middle of three PDA plates and grown for 14 days at 22 °C in the dark. Culture diameter was recorded every 3 days. Photographs were taken from the front and reverse sides of the PDA plates after 14 days of incubation. Conidia were collected with a sterile spreader after flooding the Petri plate with 2 ml sterile ddH_2_0, the spore suspension was filtered with sterile cheese cloth and microscopic slides were prepared with sterile ddH_2_O. Conidia morphology was observed using light microscopy (DM2000-LED, Leica Microsystems, Wetzlar, Germany) equipped with a high definition camera (Gryphax Subra, Jenoptik AG, Jena, Germany). A minimum of at least 50 measurements were performed to determine conidia length and width. A principal component analysis (PCA) was performed on a subset of 17 representative *C. lupini* isolates, based on average conidia length and width, length width ratio, colony growth rate, form (circular = 1, most irregular = 4), aerial mycelia (no aerial mycelia = 1, most aerial mycelia = 4), color (palest = 1, darkest = 4) and filiform margin (yes = 1, no = 0), using R 4.0.3^61^ and the *FactoMineR* package^62^.

### DNA extraction, PCR amplification and sequencing

Mycelium from single-spore cultures was collected after 7–10 days on PDA at 22 °C with a sterile spreader after flooding the Petri dish with 2 ml sterile ddH_2_0. Genomic DNA was isolated with a CTAB extraction protocol^63^. Partial gene sequences were determined for the internal transcribed spacer (ITS) region using primers ITS5 and ITS4^64^, the glyceraldehyde-3-phosphate dehydrogenase (GAPDH) gene using primers GDF1 and GDR1^65^, the β-tubulin 2 (TUB2) gene using primers Btub2Fd and Btub4Rd^66^ and the Apn2-Mat1-2-1 intergenic (APN/MAT1) spacer and partial mating type gene using Apnmat1F and Apnmat1R^39^. PCR was performed in a S1000 Thermal Cycler (Bio-Rad Laboratories, Inc., Hercules, CA, USA) according to conditions described in Dubrulle et al*.*^39^ PCR products were verified by gel electrophoresis, purified using the QIAquick PCR Purification Kit (Qiagen, Hilden, Germany), quantified with a NanoDrop ND-1000 spectrophotometer (Thermo Fisher Scientific, Waltham, MA, USA) and sent to Eurofins Genomics (Ebersberg, Germany) for sequencing. The obtained DNA sequences were analyzed and consensus sequences were generated using BioEdit v. 7.2.5^67^.

### Phylogenetic analyses

Alignments for each of the four loci, including sequences obtained in this study and downloaded from GenBank (Table 1), were performed with ClustalW using MEGA X^68^. Obtained multiple alignments where manually corrected and trimmed to obtain comparable sequences. Best-fit substitution models were determined for each locus separately and for the concatenated multi-locus alignment (ITS, TUB2, GAPDH and APN/MAT1). Phylogenetic analyses of the multi-locus alignment were based on Maximum Likelihood (ML) and Bayesian Inference (BI). The ML analysis was performed using RAxML v. 8^69^ through the CIPRES science gateway portal^70^ using default parameters and 1000 bootstrap iterations. The BI analysis was performed with MrBayes v. 3.2.7^71^ using a Markov Chain Monte Carlo (MCMC) algorithm using four chains and starting from a random tree topology. Substitution models for each locus were included for each partition. The analysis ran for 500,000 generations with trees sampled every 1000 generations to reach average standard deviations of split frequencies below 0.01. The first 25% of saved trees were discarded at the ‘burn-in’ phase and the 50% consensus trees and posterior probabilities (PP) were determined from the remaining trees. Bootstrap support values (BS) from the ML analysis were plotted on the Bayesian phylogeny. Further phylogenetic analyses were performed with the unweighted pair group method with arithmetic mean (UPGMA) with 10,000 replicates in Mega X. All generated sequences were deposited in GenBank (Table 1) and alignments and trees in TreeBASE.

### Virulence

Virulence tests were performed on white and Andean lupin with representative *C. lupini* strains (see Fig. 3), *C. tamarilloi* strain CBS 129814 and *C. acutatum* strain CBS 369.73 through stem-wound inoculation as described by Alkemade et al*.*^26^, which was shown to highly correspond to field performance in Switzerland (r = 0.95). Disease scores ranging from 1 (non-pathogenic), 2 (low virulence) to 9 (highly virulent) were taken 4, 7 and 10 days post inoculation (dpi) and the standardized area under the disease progress curve was calculated (sAUDPC)^26^. All inoculations were performed in a growth chamber (25 ± 2 °C, 16 h light and ~ 70% relative humidity) in a completely randomized block design with a minimum of six replicates per experiment.

### Statistical analysis

Statistical analyses were performed with R 4.0.3 using the packages *lme4*^72^, *lmerTest*^73^ and *emmeans*^74^, following a mixed model with factors of interest (i.e. strain, lupin species, lupin accession) as fixed and replicated block nested in experiment as random factor. Datasets that did not follow assumptions of normality of residuals and homogeneity of variance were log10 transformed. Data are presented as estimated least-squares means using the aforementioned mixed model. A Tukey-HSD test (p ≤ 0.05) was applied for pairwise mean comparison of the different *Colletotrichum* strains within each lupin accession.

## Supplementary Information

Supplementary Information.

## Data Availability

The data that support the findings of this study are shown in this manuscript or, in the case of new sequences data, are openly available in Genbank at https://www.ncbi.nlm.nih.gov/genbank/ (for reference numbers see Table 1) and in Treebase at http://purl.org/phylo/treebase/phylows/study/TB2:S27356?x-access-code=260136f8e6416a0614b93528ddbfe0ef&format=html.
