# Supplementary material for: Genetic diversity of Colletotrichum lupini and its virulence on white and Andean lupin
Source: Sci Rep. 2021 Jun 29;11:13547. doi: 10.1038/s41598-021-92953-y (PMC8242092; doi:10.1038/s41598-021-92953-y)
Supplement: Supplementary file 1 — Supplementary Information. [file 41598_2021_92953_MOESM1_ESM.pdf]

Genetic diversity of *Colletotrichum lupini* and its virulence on white and Andean lupin

J. A. Alkemade[1], M. M. Messmer[1], R. T. Voegele[2], M. R. Finckh[3], P. Hohmann[1*]

1 Department of Crop Sciences, Research Institute of Organic Agriculture (FiBL), Switzerland

2 Institute of Phytomedicine, University of Hohenheim, Germany

3 Department of Ecological Plant Protection, University of Kassel, Germany

*Corresponding author

Email: pierre.hohmann@fibl.org, Tel: +41 (0)62 865-0476

Supplementary Figure legends

Supplementary Figure S1. Principal component analysis (PCA) on strain morphology. Based on conidia length, width and length width ratio, growth rate on PDA, colony form (circular = 1, most irregular = 4), aerial mycelia (no aerial mycelia = 1, most aerial mycelia = 4), color (palest = 1, darkest = 4) and filiform (yes = 1, no = 0) of 17 *Colletotrichum lupini* strains. Strain codes are followed by genetic group.

Supplementary Figure S2. Boxplots of conidia length (A), width (B) and colony growth rate (C). Circles within boxplot indicate mean. Strain codes are followed by abbreviated country of origin and capital letters (A-J) indicating strain morphotype. Different capital letters above boxplots indicate significant differences between strains (Tuckey-HSD, $p<0.05$).

Supplementary Figure S3. Multi-locus phylogeny of *Colletotrichum lupini*. A: UPGMA tree inferred from the combined ITS, TUB2, GAPDH and APN/MAT1 sequence datasets of 50 *Colletotrichum* strains used in this study. Bootstrap support values (>50%) are given at each node. The tree is rooted to *C. acutatum* (CBS 369.73 and CBS 370.73). Strain codes are followed by host, country of origin and morphology (A-J). Grouping is based phylogeny and morphology. Strains used for virulence assays are highlighted in bold.

Supplementary Figure S4. Representative plants showing disease symptoms of *Colletotrichum lupini* strains on white (*Lupinus albus)* and Andean lupin (*L. mutabilis*). Lupin seedlings of the different species and accessions (Feodora, Blu-25, LUP 17, LUP 100) were stem inoculated with different *C. lupini* strains 14 days after sowing. Photos and disease score indices (DSI; top right corners) were taken 10 days post-inoculation. Strain codes are followed by country of origin, genetic group and morphotype.

Supplementary Table S1: Identical sites (%) across the nucleotide dataset per locus. CaSC indicates variability within *Colletotrichum acutatum* species complex. *C. lupini* indicates variability within *C. lupini.*

	**CaSC**	***C. lupini***
**ITS**	97	99.2
**GAPDH**	81.1	98.4
**TUB2**	89.9	97.8
**APN/MAT1**	75.8	97.4

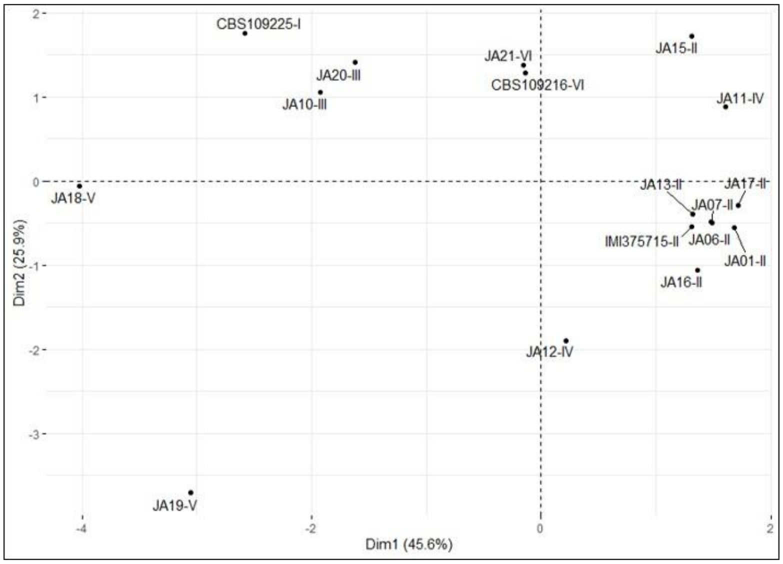

Supplementary Figure S1. Principal component analysis (PCA) on strain morphology. Based on conidial length, width and length width ratio, growth rate on PDA, colony form (circular = 1, most irregular = 4), aerial mycelia (no aerial mycelia = 1, most aerial mycelia = 4), color (palest = 1, darkest = 4) and filiform (yes = 1, no = 0) of 17 *Colletotrichum lupini* strains. Strain codes are followed by genetic group.

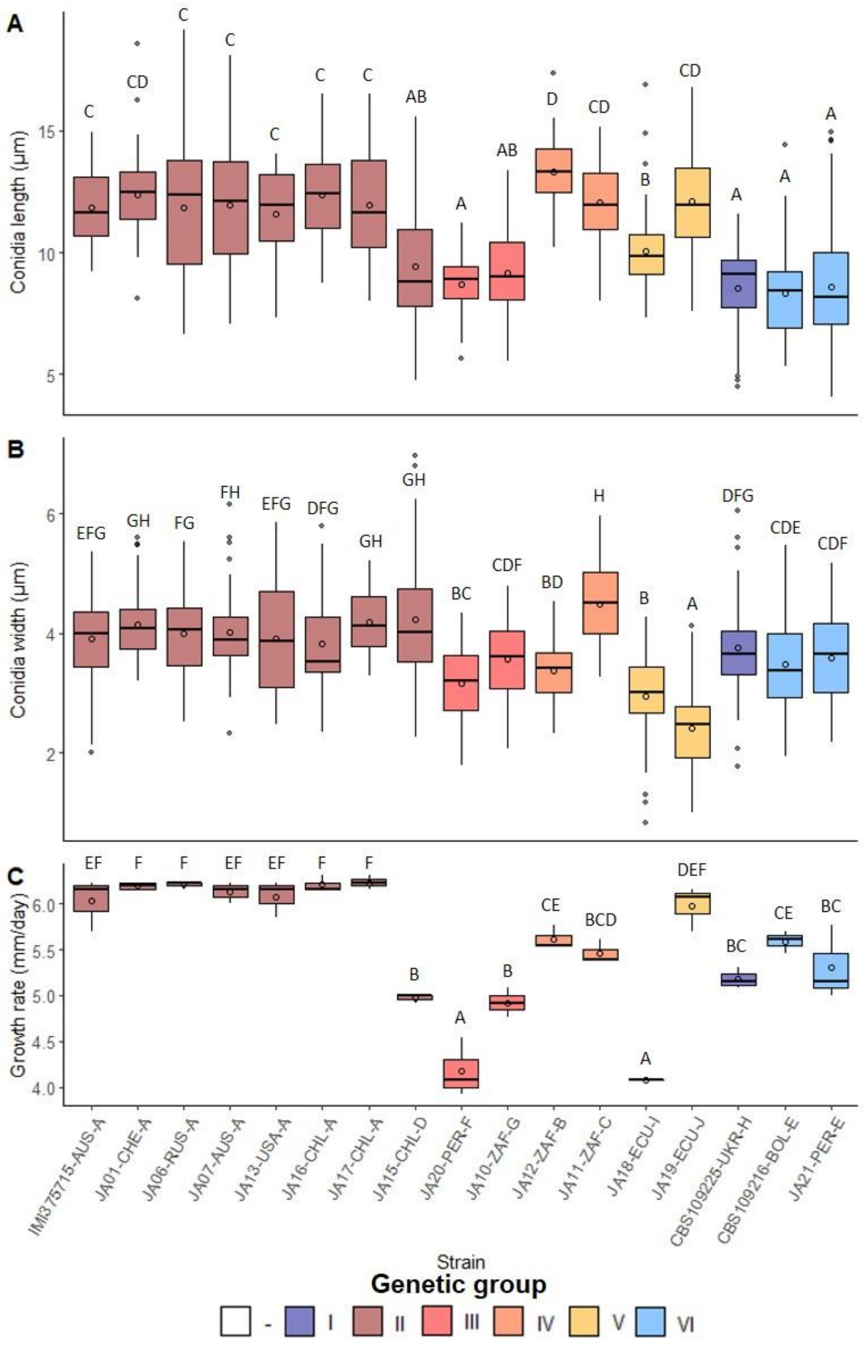

Supplementary Figure S2. Boxplots of conidia length (A), width (B) and colony growth rate (C). Circles within boxplot indicate mean. Strain codes are followed by abbreviated country of origin and capital letters (A-J) indicating strain morphotype. Capital letters above boxplots indicate significant differences between strains (Tuckey-HSD, p<0.05).

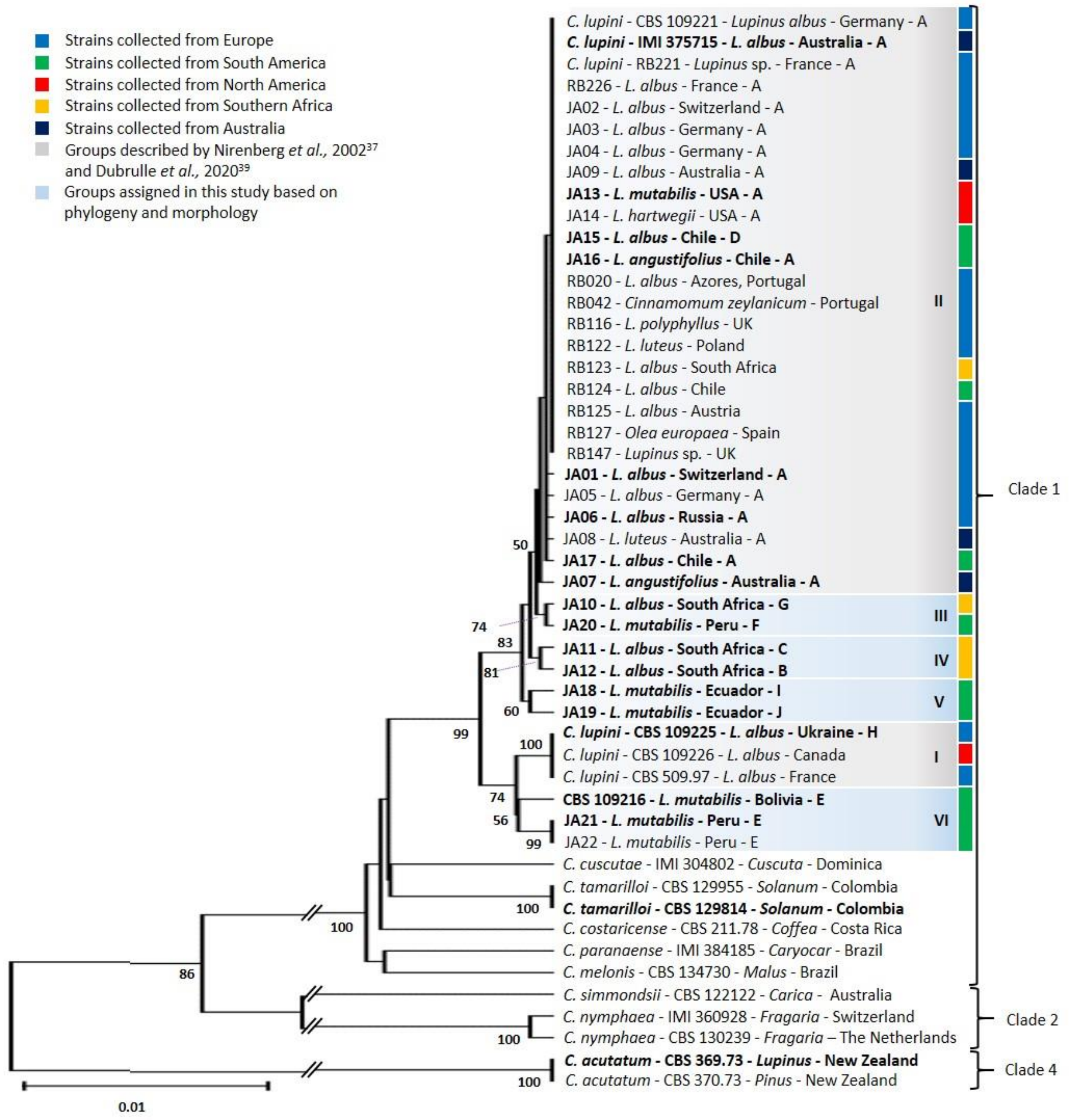

Supplementary Figure S3. Multi-locus phylogeny of *Colletotrichum lupini*. UPGMA tree inferred from the combined ITS, TUB2, GAPDH and APN/MAT1 sequence datasets of 50 *Colletotrichum* strains used in this study. Bootstrap support values (>50%) are given at each node. The tree is rooted to *C. acutatum* (CBS 369.73 and CBS 370.73). Strain codes are followed by host, country of origin and morphology (A-J). Grouping is based phylogeny and morphology. Strains used for virulence assays are highlighted in bold.

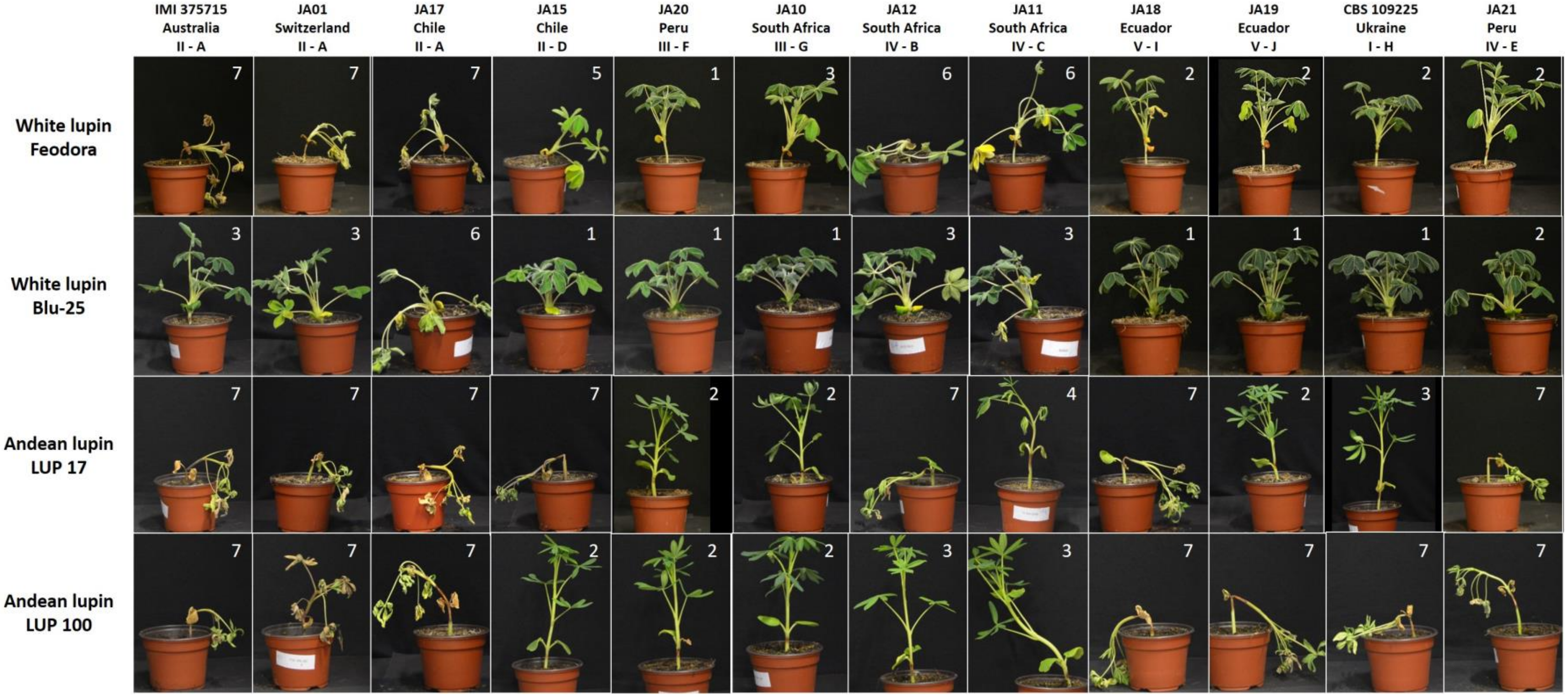

Supplementary Figure S4. Representative plants showing disease symptoms of *Colletotrichum lupini* strains on white (*Lupinus albus)* and Andean lupin (*L. mutabilis*). Lupin seedlings of the different species and accessions (Feodora, Blu-25, LUP 17, LUP 100) were stem inoculated with different *C. lupini* strains 14 days after sowing. Photos and disease score indices (DSI; top right corners) were taken 10 days post-inoculation. Strain codes are followed by country of origin, genetic group and morphotype.
